# Kennedy’s disease presented with mastication fatigue combined with positive titin antibody: a case report

**DOI:** 10.1186/s12883-022-02971-0

**Published:** 2022-11-14

**Authors:** Guang Ji, Rui Huang, Xiaomeng Zhou, Cuifang Cao, Qiong Wu, Zhenfei Li, Hui Dong, Xueqin Song, Yaling Liu

**Affiliations:** 1 Department of Neurology, The Second Hospital of Hebei Medical University, 050000 Shijiazhuang, Hebei People’s Republic of China; 2 Neurological Laboratory of Hebei Province, 050000 Shijiazhuang, Hebei People’s Republic of China; 3 Department of Cerebrovascular Disease and Spine Neurosurgery, The People’s Hospital Of Guangxi Zhuang Autonomous Region, 530016 Nanning, People’s Republic of China

**Keywords:** SBMA, Mastication fatigue, Repetitive nerve stimulation, Titin antibody, WPW syndrome

## Abstract

**Background:**

Spinal and bulbar muscular atrophy (SBMA) is an X-linked recessive hereditary neuromuscular disorder caused by the expanded trinucleotide repeat in the androgen receptors gene. The major clinical manifestations of SBMA consist of weakness in the bulbar and limb muscles, fasciculations, tremors, cramps, sensory impairment, and gynecomastia. However, atypical SBMA cases may lead to misdiagnosis. Muscular fatigue and decremental responses to repetitive nerve stimulation (RNS), despite being observed in some SBMA patients, are usually occurred in MG patients, and patient with the symptom of mastication fatigue was rarely reported. In addition, cardiological investigations have been performed in SBMA patients and several ECG alterations were identified. Here we report an SBMA patient presenting with a rare onset symptom of mastication fatigue, who has been detected with a positive titin antibody in the serum and showed a WPW pattern electrocardiogram.

**Case presentation:**

The patient showed mildly progressive fatigue in the muscles of mastication over 3 years. Neurological examination showed facial muscle weakness and a wasting tongue with fasciculations, but the weakness, wasting, or fasciculations were not obvious in the limbs. 3-Hz RNS showed a decremental response in bilateral orbicularis oculi. The test of titin antibody was positive in the serum, and the electrocardiogram showed a WPW pattern ECG. Genetic analysis revealed an increased number (39 repeats) of tandem CAG repeats in the AR gene, which confirmed the diagnosis of SBMA. The fatigue symptom was significantly improved after oral pyridostigmine bromide treatment.

**Conclusion:**

This case calls for more attention to muscular fatigue as the onset symptoms of Kennedy’s disease. ECG screening is of importance in SBMA patients and further studies are needed to investigate the titin antibody in SBMA patients as well as other neurogenic disorders.

**Supplementary Information:**

The online version contains supplementary material available at 10.1186/s12883-022-02971-0.

## Background

Spinal and bulbar muscular atrophy (SBMA), also called Kennedy’s disease, is an X-linked recessive hereditary neuromuscular disorder characterized by adult onset, slowly progressive weakness, and atrophy of proximal limbs and bulbar muscles [1]. This disease is caused by the expanded trinucleotide repeat (CAG > 37) in the androgen receptors (AR) gene which encodes glutamine [2]. The toxicity of the polyglutamine-expanded AR accumulation leads to multisystem involvement.

The main clinical manifestations of SBMA consist of weakness in the bulbar and limb muscles, fasciculations, tremors, cramps, sensory impairment, gynecomastia, as well as sexual dysfunction. However, atypical SBMA cases were also reported, which may lead to misdiagnosis. In previous studies, muscular fatigue and decremental responses to repetitive nerve stimulation (RNS) were observed in some patients with Kennedy’s disease [3–6], which more frequently occurred in Myasthenia Gravis (MG). Non-neural manifestations are also present in SBMA patients, such as gynecomastia, sexual dysfunction, metabolic abnormalities, and myocardial involvement. Different ECG alterations have been observed in SBMA patients [7–9]. Here we report an SBMA patient presenting with a rare onset symptom of mastication fatigue, who has been detected with WPW pattern ECG. Interestingly, we also detected a positive titin antibody in the serum of this patient. This case expands the understanding of the clinical manifestations and the pathophysiological changes in Kennedy’s disease.

## Case presentation

A 52-year-old male manifested mildly progressive fatigue in the muscles of mastication over 3 years, without diplopia, dysphagia, or limb weakness. The patient complained of fatigue when chewing hard food (beef jerky, dried sweet potatoes, pancakes, etc.), but not obvious when eating soft food (rice, noodles, etc.). He also felt easy to cramp in the muscles of the neck, abdomen, and limbs. During the 3 years, he felt the symptoms slightly aggravated. Additionally, the patient suffered from hypertension for 6 years and pre-excitation syndrome for 5 years and accepted surgery because of a kidney stone 8 years ago (Table 1). A younger brother of the patient presented a postural tremor in his hands for 10 years.

**Table 1:** The timeline with relevant data from the episode of care

Time	Episode of care	Examination and treatment
2014	The patient was diagnosed as a pre-excitation syndrome	none
2016	Progressive fatigue in the muscles of mastication, easy cramp in the muscles of the neck, abdomen, and limbs	none
2019	Visit our hospital	Electrophysiology examination, laboratory tests and gene analysis were performed, and the patient received oral pyridostigmine bromide

Neurological examination revealed reduced strength of masticators after repeated movements. Weakness and spasms of facial muscles were present, and tongue wasting with fasciculations was observed (Video 1). While the weakness, wasting, or fasciculations were not obvious in the limbs. The patient also showed slightly postural hand tremor, and ptosis in the right eyelid was observed. Muscle strength in his upper and lower limbs was 5/5 according to the Medical Research Council score, and the Quantitative Myasthenia Gravis score was 4 (3 in ptosis, 1 in facial muscles). Muscle reflexes and muscle tone were normal. Sensation and coordination were intact. There was no gynecomastia.

**Fig. 1:**
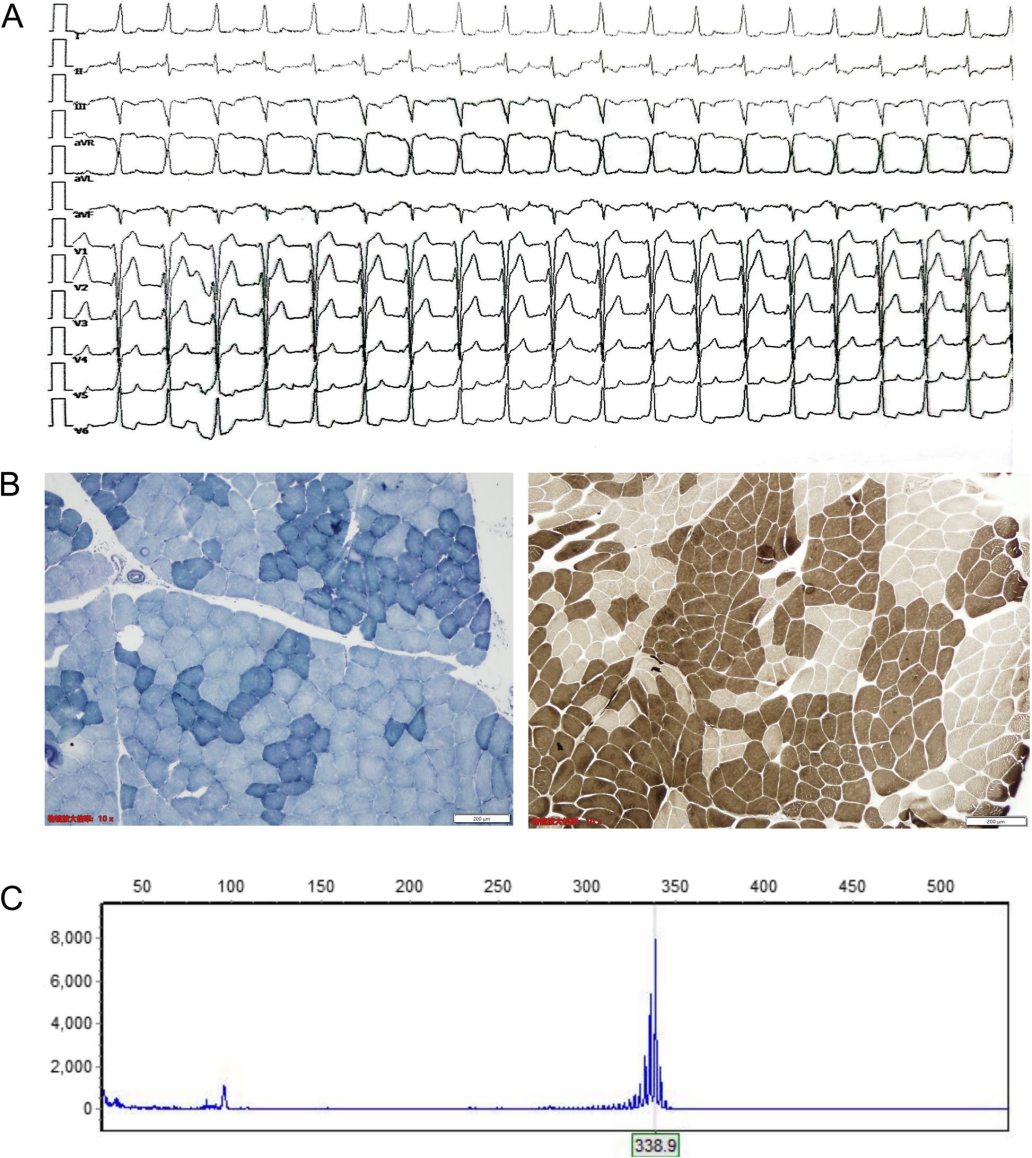
** A** The Electrocardiograph of this patient showed a Wolf-Parkinson-White (WPW) pattern ECG with a heart rate of 80 bpm. **B** the muscle pathology showed a grouping muscle fibers in NADH-TR and ATPase (pH10.2) staining. **C** Fragment length analysis of the patient revealed an increased number (39 repeats) of tandem CAG repeats in the AR gene

Routine laboratory tests showed a normal creatine kinase level but detected an elevated level of serum uric acid (501umol/L, normal range 208-428umol/L) and triglyceride (4.53mmol/L, normal range 0.56-1.70mmol/L). Thyroid function and sex hormones were normal. The lactic acid stress test was negative. Tests of AchR antibody (ELISA), MUSK antibody (CBA), LRP4 antibody (ELISA), and RyR antibody (ELISA) were normal, while the test of titin antibody (ELISA) was positive (1.09 OD). The electrocardiogram showed a WPW pattern ECG with a heart rate of 80 bpm (Fig. 1A), but the patient did not manifest any cardiological symptoms such as palpitation. CT scans of the lung and thymus gland were normal. Brain MRI showed supratentorial multiple ischemic changes.

Nerve conduction studies showed decreased action potentials in the right median, ulnar and bilateral sural sensory nerves, and the left peroneal compound muscle action potential was lower in amplitude than the right side. Both motor and sensory nerve conduction velocities were normal. Needle electromyographic studies showed high-amplitude, long-duration motor unit potentials in a diffuse distribution of limbs, tongue, rectus abdominis, paravertebral, and sternocleidomastoideus muscles, and showed simple phases in all tested muscles. Fibrillation and positive sharp waves were only observed in the tongue muscle. The above results revealed chronic neurogenic damage with both motor and sensory involvement. In addition, the 3-Hz RNS showed a decremental response in bilateral orbicularis oculi (14.9% on the right side and 12% on the left side) but did not detect a decremental response in trapezius or abductor digiti minimi, and no significant decremental or incremental response to 30-Hz RNS was noted.

After informed consent, a muscle biopsy was performed on his left biceps brachii, and the muscle pathology showed a grouping muscle fibers, indicating chronic neurogenic damage (Fig. 1B). Genomic DNA was obtained from the peripheral blood leukocytes. The genetic analysis was performed using polymerase chain reaction and fragment length analysis, which revealed an increased number (39 repeats) of tandem CAG repeats in the AR gene confirming the diagnosis of SBMA (Fig. 1C). The same genetic analysis result was also detected in his brother. The patient felt the fatigue symptom significantly improved after oral pyridostigmine bromide treatment (30 mg, Tid), but the symptoms keep progressing in the subsequent follow-up process.

## Discussion and conclusions

Spinal and bulbar muscular atrophy (SBMA) is a rare, late-onset, X-linked hereditary disease caused by CAG trinucleotide abnormal expansion in the androgen receptor (AR) gene. Bulbar, facial, and proximal limb muscles are frequently affected in this disease, but atypical SBMA cases may often lead to misdiagnosis. The patient in this case presented with mastication fatigue as the onset symptom. We detected abnormal decremental responses in this patient, and he exhibited a significant improvement in symptoms after oral administration of pyridoxamine bromide in the follow-up treatment. The above manifestation usually occurs in MG. Actually, despite as a kind of motor neuronopathy, SBMA has been reported to show myasthenic symptoms or decremental responses to RNS in many cases, and positive responses to acetylcholinesterase inhibitors have also been described [3, 6, 10]. In previous studies, abnormal decremental responses were detected most commonly in the trapezius muscle in SBMA, while on the other hand, the affected ALS patients showed decremental responses mainly in the distal hand muscles [4, 5]. Research showed the decremental response in SBMA might be attributed to the toxicity of the expanded AR accumulation on muscles or neuromuscular junction [11], or the motor neurons in SBMA may have a reduced capacity for reinnervation. Besides, the reduced capacity for reinnervation in the trapezius muscle could be accounted for by the greater size of muscle fibers and lower fiber density in the trapezius muscle [5].

Interestingly, although the test for AchR antibody, MUSK antibody, and LRP4 antibody was negative, the titin antibody was positive in the serum of this patient. Skeletal muscle titin is known as the largest human protein functioning in the connection of the Z-disc to the M-line in the sarcomere. Research has shown that titin antibodies in patients with early-onset MG can suggest a possibility of the presence of thymoma and indicate a more severe type of MG [12]. Even if studies revealed titin antibodies as a valuable biomarker for MG diagnosis, intracellular localization of titin protein makes it unlikely for the corresponding antibodies to have a direct pathogenic role in MG [13]. Our patient showed no thymoma and did not receive immunological therapy, thus whether the positive titin antibody is responsible for fatigue symptoms remains unclear. Titin is cleaved by calpain-3 during muscular damage, resulting in the release of an N- terminal fragment into the urine via glomerular filtration, and recent research have found that urinary levels of titin N-terminal fragment were elevated in ALS as well as in other neurogenic disorders, including SBMA [14]. It is supposed that the titin fragmentation was induced by muscular damage associated with motor neuron degeneration, which caused titin cleavage without an overt leakage of CK. Based on these findings, we hypothesize the titin antibody detected in our patient may be stimulated by the systematic aberrant accumulation and antigen exposure of titin fragments during the muscle damage. Large sample studies were needed to investigate the titin antibody in SBMA patients as well as other neurogenic disorders. Furthermore, titin antibodies were also detected in some biopsy-proven inflammatory myopathy with myasthenia gravis patients, and these patients frequently show polymyositis pathology [15], indicating a relationship between inflammatory pathology and anti-titin antibodies. However, we did not observe any inflammatory findings except for obvious neurogenic damage features in the muscle pathology of this patient, which also support that titin antibodies were produced secondary to muscular damage associated with motor neuron degeneration.

In addition to symptoms of neuromuscular involvement, many non-neural manifestations may be present in the SBMA patients, such as gynecomastia, metabolic disturbances, and myocardial involvement. Studies have been made to detect subtle myocardial abnormalities in patients with SBMA. A Japanese study indicates that a Brugada-like electrocardiogram was the most common feature [7]. Other ECG alterations were also identified, including ST-segment abnormalities, early repolarization, fragmented QRS, pathological Q-waves, and intraventricular conduction abnormalities [7–9]. The WPW pattern ECG has not been reported in previous studies and was detected in our case for the first time. The mechanisms under the ECG alterations in SBMA patients were still unclear. It was found that the SCN5A gene, which was associated with Brugada syndrome, showed a downregulated expression in the myocardium of patients with SBMA [7], indicating that the gene expression abnormalities result from the nuclear accumulation of pathogenic AR may be responsible for the myocardial dysfunction in SBMA. It has been previously found that WPW syndrome was associated with mutation in AMP-activated protein kinase (AMPK) subunit genes [16, 17], and the expression of these genes may also be affected by the extensive nuclear accumulation of pathogenic AR. We speculate that the WPW pattern ECG was caused by the same mechanism as that in SBMA patients with Brugada syndrome. Moreover, this myocardial abnormality occurred earlier than the motor symptoms, indicating that the heart may be an early involved organ in Kennedy’s disease.

Metabolic disturbances are also found in Kennedy’s disease, such as elevated total cholesterol, low-density lipoprotein, and triglycerides [18]. Insulin resistance and fatty liver were also reported in many SBMA patients [19]. However, the underlying mechanism of metabolic alterations in SBMA remains unclear. Testosterone and androgen receptors are of great importance in the regulation of insulin signaling and other aspects of metabolic syndrome, so the alteration of AR function and androgen insensitivity may contribute to fatty liver and insulin resistance [19]. In addition to the elevated level of triglyceride, we also detected an elevated level of serum uric acid in this patient, which may also be a metabolic disturbance associated with SBMA or just a coincidence.

In summary, we report an SBMA patient with an onset symptom of mastication fatigue, who was found with a positive titin antibody and a WPW pattern ECG. This case calls for more attention to muscular fatigue as the onset symptoms of Kennedy’s disease to avoid the missed diagnosis and expands the understanding of the pathophysiological changes in SBMA. Further studies are needed to investigate the mechanism of the positive antibody and EEG alterations in Kennedy’s disease.

## Supplementary Information

**Additional file 1:** **Video 1.** Tongue wasting with fasciculations was observed in this patient.

## Data Availability

The datasets for this article are not publicly available due to participant/patient anonymity concerns. Requests to access the datasets should be directed to the corresponding author.

## Abbreviations

SBMA: Spinal and bulbar muscular atrophy
AR: Androgen receptors
RNS: Repetitive nerve stimulation
WPW: Wolf-Parkinson-White
MG: Myasthenia Gravis
ECG: Electrocardiograph
ELISA: Enzyme-linked immunosorbent assay
CBA: Cell-based assay
